# A real-time traffic control method for the intersection with pre-signals under the phase swap sorting strategy

**DOI:** 10.1371/journal.pone.0177637

**Published:** 2017-05-22

**Authors:** Yiming Bie, Zhiyuan Liu, Yinhai Wang

**Affiliations:** 1 School of Transportation Science and Engineering, Harbin Institute of Technology, Harbin, China; 2 Jiangsu Key Laboratory of Urban ITS, Jiangsu Province Collaborative Innovation Center of Modern Urban Traffic Technologies, Southeast University, China; 3 Department of Civil and Environmental Engineering, University of Washington, Seattle, Washington, United States of America; Beihang University, CHINA

## Abstract

To deal with the conflicts between left-turn and through traffic streams and increase the discharge capacity, this paper addresses the pre-signal which is implemented at a signalized intersection. Such an intersection with pre-signal is termed as a tandem intersection. For the tandem intersection, phase swap sorting strategy is deemed as the most effective phasing scheme in view of some exclusive merits, such as easier compliance of drivers, and shorter sorting area. However, a major limitation of the phase swap sorting strategy is not considered in previous studies: if one or more vehicle is left at the sorting area after the signal light turns to red, the capacity of the approach would be dramatically dropped. Besides, previous signal control studies deal with a fixed timing plan that is not adaptive with the fluctuation of traffic flows. Therefore, to cope with these two gaps, this paper firstly takes an in-depth analysis of the traffic flow operations at the tandem intersection. Secondly, three groups of loop detectors are placed to obtain the real-time vehicle information for adaptive signalization. The lane selection behavior in the sorting area is considered to set the green time for intersection signals. With the objective of minimizing the vehicle delay, the signal control parameters are then optimized based on a dynamic programming method. Finally, numerical experiments show that average vehicle delay and maximum queue length can be reduced under all scenarios.

## Introduction

### Background

If the traffic volume at one signalized intersection exceeds its capacity, the consequent traffic congestion can significantly increase the vehicle delay, stop rate and fuel consumption of each driving commuter. The conflicts between left-turn vehicles and their opposite through vehicles are the most important factor in the congestion here. Since little improvement can be made on the signal control scheme (e.g. green split, cycle length), the optimization of lane assignment thus becomes a good strategy to deal with these conflicts. Simply converting one or more through lane to left-turn lanes can increase the left-turn capacity, but the capacity of through lanes is largely reduced, and vice versa. Therefore, a few strategies to regulate the left-turn vehicles at intersections are proposed, e.g. U turn [1, 2, 3], Hook turn [4, 5, 6], displaced left turn [7], where the main idea is to remove the left-turn phase or reduce the left-turn green time to improve the overall capacity of the intersection. Under such strategies, however, the left-turn vehicles and conflicts are shifted to the nearby road segments. Furthermore, these strategies have certain special requirements on the geometry of intersections.

**Fig 1:**
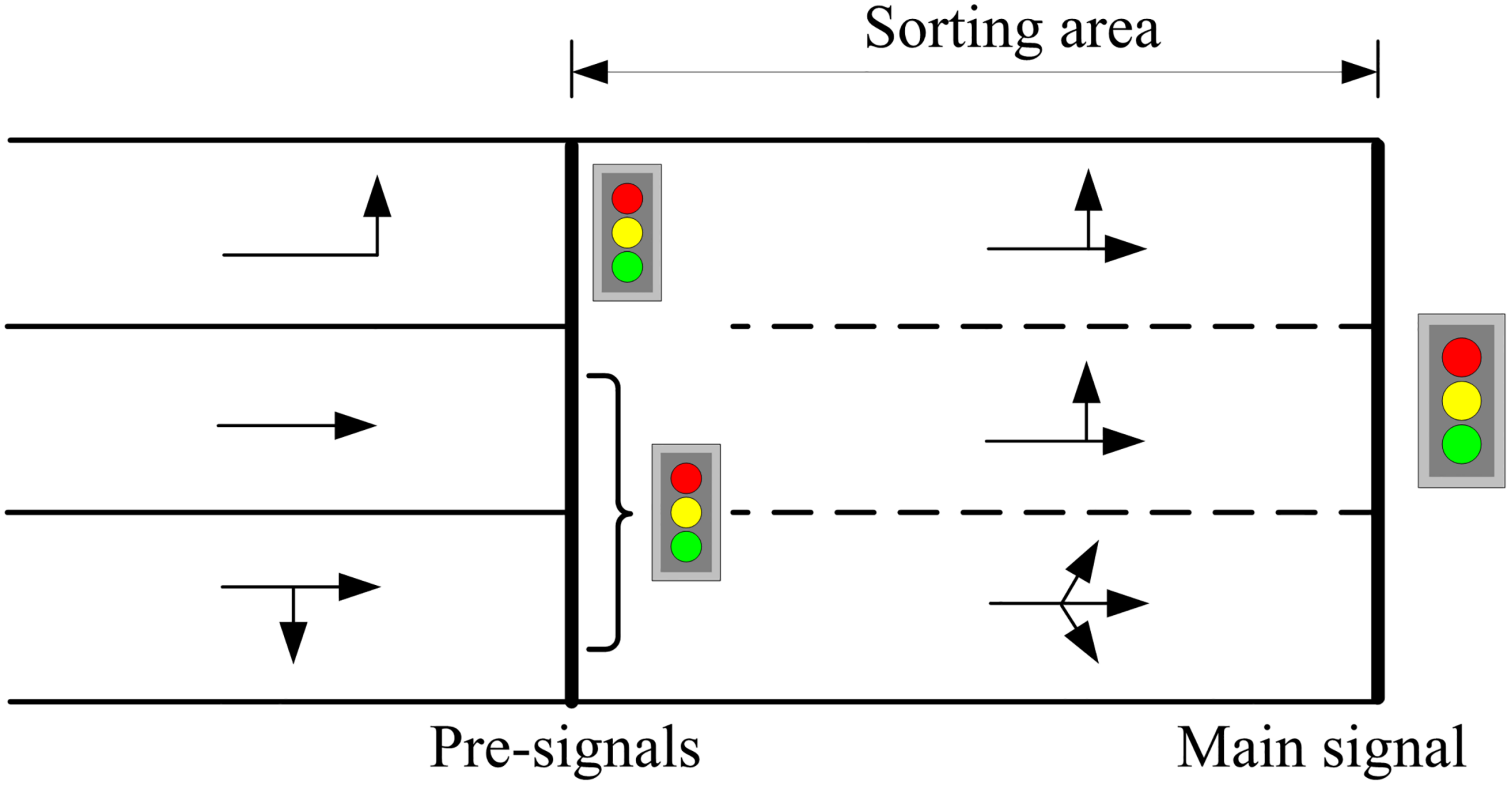
The pre-signal on an approach of a tandem intersection.

A new method was proposed recently where an additional stop line and signal is set at the upstream of the intersection. This newly-set signal is defined as the *pre-signal* while the original one is the main signal. The area between the two signals is termed the sorting area. Fig 1 shows the geometrical layout of an intersection with pre-signal, termed as tandem intersection. Two signal groups are set at the pre-signal for controlling left-turn and through vehicles respectively. When the pre-signal turns green, vehicles are allowed to enter and wait in the sorting area, and pass the intersection once the main signal turns green. Compared with the traditional methods for intersection design, pre-signals increase the number of lanes available for both left-turn and through vehicles and thus, significantly improve the intersection capacity. For example in Fig 1, three lanes can be used by left-turn vehicles whereas only one is available in the traditional intersection design.

**Fig 2:**
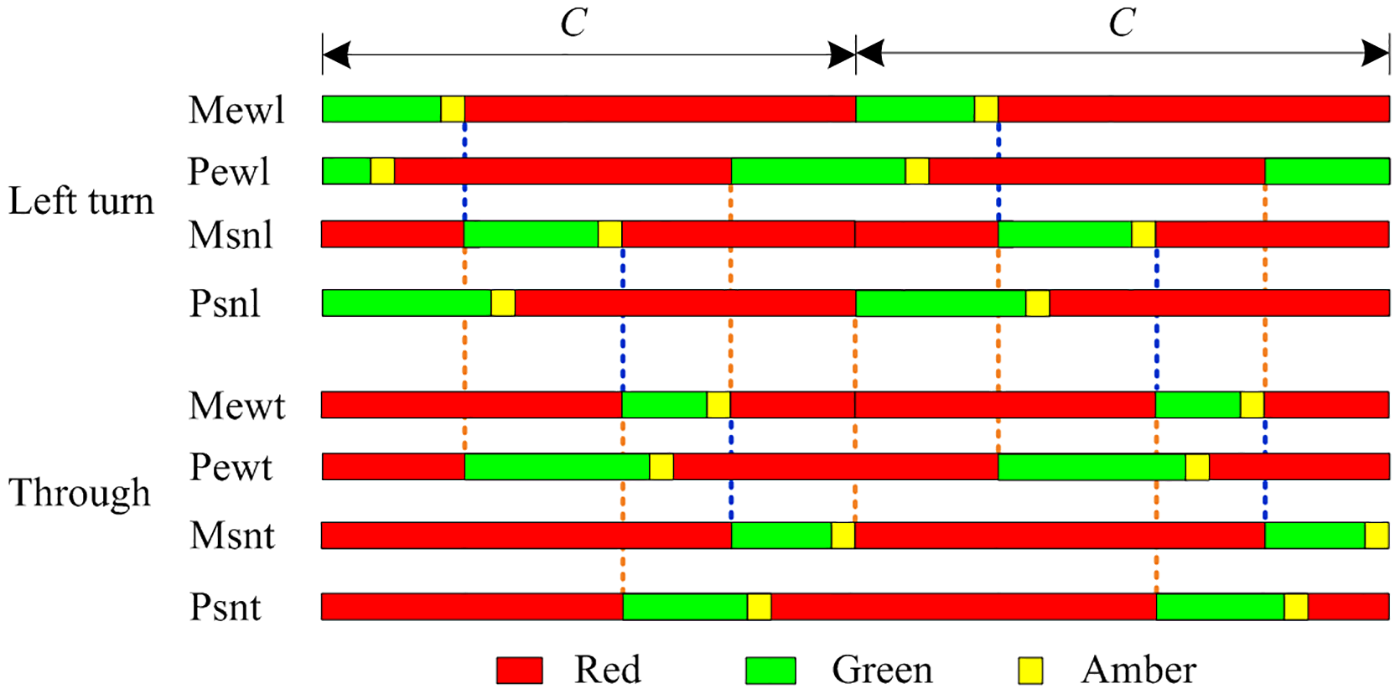
Phasing scheme for the intersection with pre-signals proposed by Yan et al.

A critical component of traffic control at the tandem intersection is the phasing scheme design, which directly determines the operation rules of traffic flow, and also influences the lane design and the optimization of signal timing parameters. For a four-approach intersection with fully-installed pre-signals (one for each approach), Yan et al. [8] proposed a phase swap sorting strategy for designing the phasing scheme and it is shown in Fig 2. We define Mewl and Mewt (Msnl and Msnt) as the left-turn and through phases of the main signal on the eastbound and westbound (southbound and northbound) arms, and Pewl and Pewt (Psnl and Psnt) as the left-turn and through phases of the pre-signal on the eastbound and westbound (southbound and northbound) arms.

Hence the explicit operation rules of the phase swap sorting strategy are:

1. When Pewl turns green, left-turn vehicles queuing behind the pre-signal stop lines from the eastbound and westbound arms enter the sorting areas, and move through the intersection once Mewl turns green. To prevent vehicles from being delayed in the sorting areas, the end time of Pewl’s green phase should precede that of Mewl. When the green phase of Mewl ends, Pewt turns green, i.e. through vehicles queuing behind the pre-signal stop lines from the eastbound and westbound arms are allowed to enter the sorting areas.
2. During the green phase of Mewl, Psnl turns green allowing let-turns vehicles to enter the sorting areas. When the green phase of Mewl ends, Msnl turns green and thus, left-turn vehicles traveling southbound and northbound move through the intersection. It is required that the end time of Psnl’s green phase precede that of Msnl. When the green phase of Msnl ends, Psnt turns green so that through vehicles queuing behind the pre-signal stop lines from the southbound and northbound arms enter the sorting areas.
3. During the green phase of Msnl, Pewt turns green allowing through vehicles to enter the sorting areas. When the green phase of Msnl ends, Mewt turns green and thus, through vehicles traveling eastbound and westbound move through the intersection. It is required that the end time of Pewt’s green phase precede that of Mewt. When the green phase of Mewt ends, Psnt turns green so that left-turn vehicles queuing behind the pre-signal stop lines from the eastbound and westbound arms enter the sorting areas.
4. During the green phase of Mewt, Psnt turns green allowing through vehicles to enter the sorting areas. When the green phase of Mewt ends, Msnt turns green and thus, through vehicles traveling southbound and northbound move through the intersection. It is required that the end time of Psnt’s green phase precede that of Msnt. When the green phase of Msnt ends, Psnl turns green so that left-turn vehicles queuing behind the pre-signal stop lines from the southbound and northbound arms enter the sorting areas.
5. During the green phase of Msnt, Pwel turns green so that left-turn vehicles traveling eastbound and westbound are allowed to enter the sorting areas.

By repeating the above five steps, a complete phasing scheme is obtained for the tandem intersection. However, we can still find some drawbacks in this phasing scheme. If a left-turn vehicle is delayed and stays in the sorting area when the green phase of Msnl ends, for example, through vehicles cannot use and queue in the lane occupied by the left-turn vehicle (because this lane is blocked when the Msnt is on) and hence, the capacity of the intersection is largely reduced.

Previous studies mainly focused on optimizing the signal timing plan for the tandem intersection based on fixed-time control strategies, without consideration of both the capacity drop phenomenon led by delayed vehicles in the sorting areas and the random disturbance of traffic flow. Therefore, to further improve the operation efficiency of traffic, this paper proposes a layout scheme for loop detectors at the tandem intersection to obtain real-time information of traffic flow and to establish a dynamic approach for traffic control.

### Literature review

**Fig 3:**
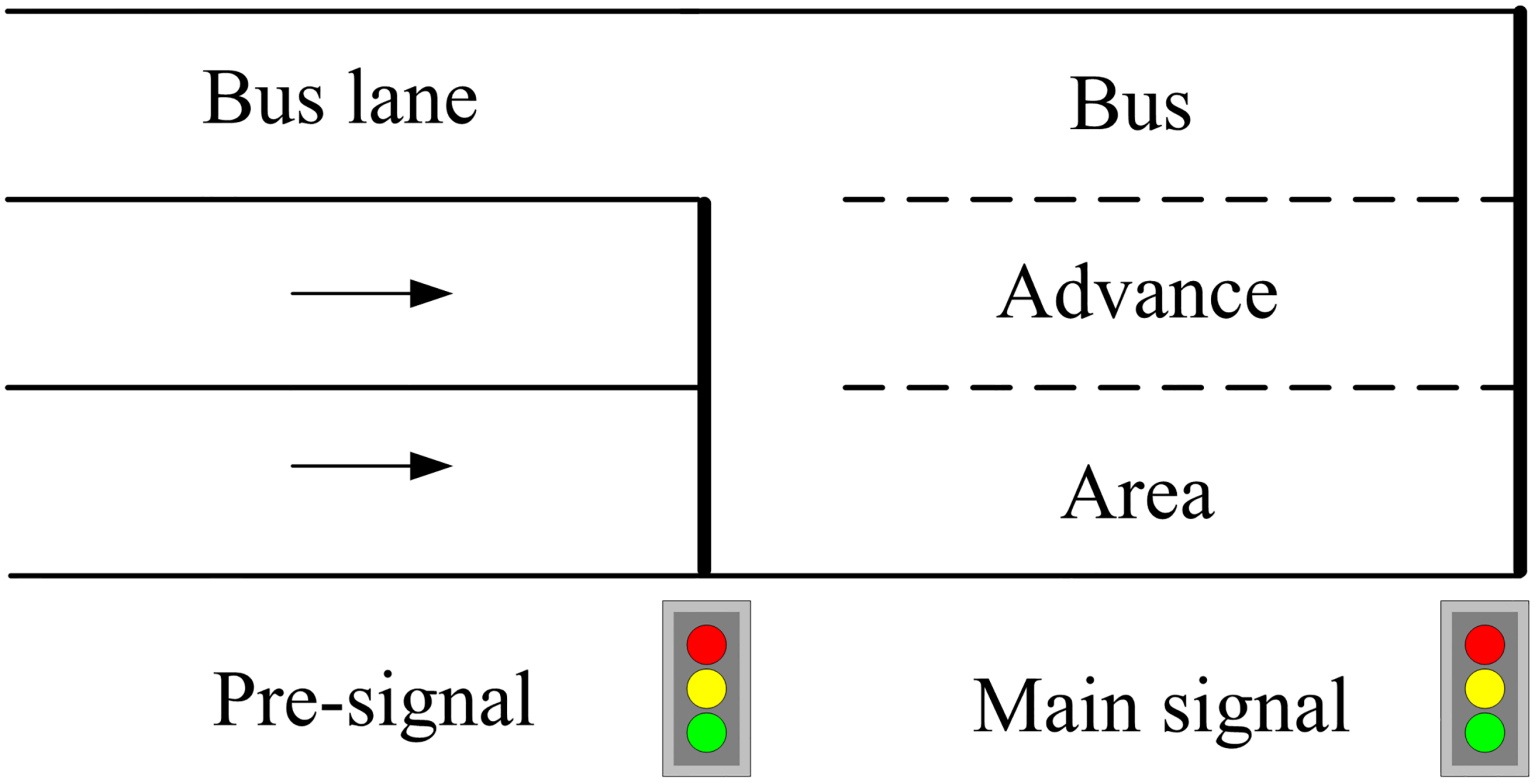
Bus pre-signal at a signalized intersection.

The concept of the tandem intersection develops from the bus pre-signal which is a type of transit priority measure. To reduce transit delays, the bus pre-signal allows transit vehicles to queue ahead of private vehicles and move through the intersection once the signal turns green [9]. Fig 3 illustrates a typical intersection with the bus pre-signal installed, where transit vehicles are allowed to directly enter the bus advance area whereas private vehicles are withheld behind the pre-signal stop line. The pre-signal turns green before the main signal allowing private vehicles to enter the bus advance area but queue behind transit vehicles. Hence priority is given to transit once the main signal turns green. Both analytical investigations and field implementations of pre-signals provide evidence that they can yield significant delay savings to buses while adding only modest travel delays to cars [10–14]. This strategy has been implemented in several countries, including the U.K., Switzerland and Germany.

Recently the bus pre-signal method is investigated by some researchers to organize the traffic streams (not only buses) at an intersection, which can improve the overall capacity of the intersection. Fig 1 shows such kind of tandem intersection. Xuan et al. [15] brought forward a novel method to eliminate the left-turn phase by reorganizing traffic on all the lanes upstream of an intersection using a mid-block pre-signal. Then it was proved that the intersection could be improved significantly, even if drivers behaved randomly and only one lane was reorganized. He also analyzed the capacity drop phenomenon caused by the delayed vehicles in the sorting area. Ma et al. [16] developed and evaluated a coordinated optimization model for an isolated intersection approach with pre-signals to increase protected left turn phase capacity. The pre-signal model was developed by capturing the interaction between the pre-signal signal and the main-signal. Yan et al. [8] developed a capacity optimization model for an isolated intersection with pre-signal based on the work of Xuan et al. The phase swap sorting strategy was implemented and all through, left- and right-turning movements on all arms were taken into consideration. Zhou and Zhuang [17] considered the extra delay aroused by the coordination, and a lane assignment and signal timing optimization model is developed to minimize the vehicle delay. The model is reformed to a mixed-integer non-linear programming.

The majority of the existing research on the tandem intersection focused on analyzing the impacts of control parameters and random disturbance on the performance indicators such as the intersection capacity and delay. However, the following two gaps are observed:

1. Existing methods for signal control are fixed-time, which cannot be adaptive to the fluctuations of traffic flows. Also the capacity drop phenomenon caused by the delayed vehicles in the sorting area is not considered, which undermines the practical implementation of signal control. Under the fixed-time control strategy, signal controller is not able to obtain the real-time information of traffic flow and hence, thus cannot dynamically detect the traffic conditions within the sorting area as well as the capacity drop. Though Xuan et al. [15] analyzed the capacity drop caused by the delayed vehicles, no solution was brought forward.
2. The queuing process within the sorting area is critical to the optimization of signal timing at the intersection. According to field observations, the lane selection behavior of left-turn or through vehicles is affected by the number of existing queuing vehicles in each lane and their distance to the end of each queue. As a result, it’s problematic for the existing studies to assume that vehicles are evenly distributed across each lane, which is obviously inconsistent with the practical experience and may lead to a suboptimal timing plan.

### Objectives and contributions

This study aims to fill the above two gaps, targeting at a four-approach intersection with fully-installed pre-signals (one for each approach). By setting multiple groups of loop detectors at the intersection, we aim to address the issue of capacity drop caused by delayed vehicles in the sorting area using real-time monitoring. The optimization objective is to minimize the average delay at the intersection by establishing a dynamic signal control method that overcomes the existing drawbacks of fixed-time control.

The major contributions of this study are twofold: (i) An adaptive signal control method is firstly developed for the tandem intersection, which can avoid the capacity drop caused by the delayed vehicles in the sorting area, and adjust the timing parameters according to real-time traffic flow information. (ii) The lane selection behavior within the sorting area is analyzed and a lane selection model is proposed, which are critical in determining the length of the sorting area and the green time of the main signal.

The remainder of this paper is organized as follows. Section 2 proposes the real-time signal control method for the tandem intersection under the phase swap sorting strategy, consisting of the layout scheme of loop detectors, the queuing process within the sorting area, and the signal control model. Section 3 evaluates the proposed signal control method through numerical tests. Section 4 concludes the paper.

## Real-time signal control method

### Layout of inductive loop detectors

**Fig 4:**
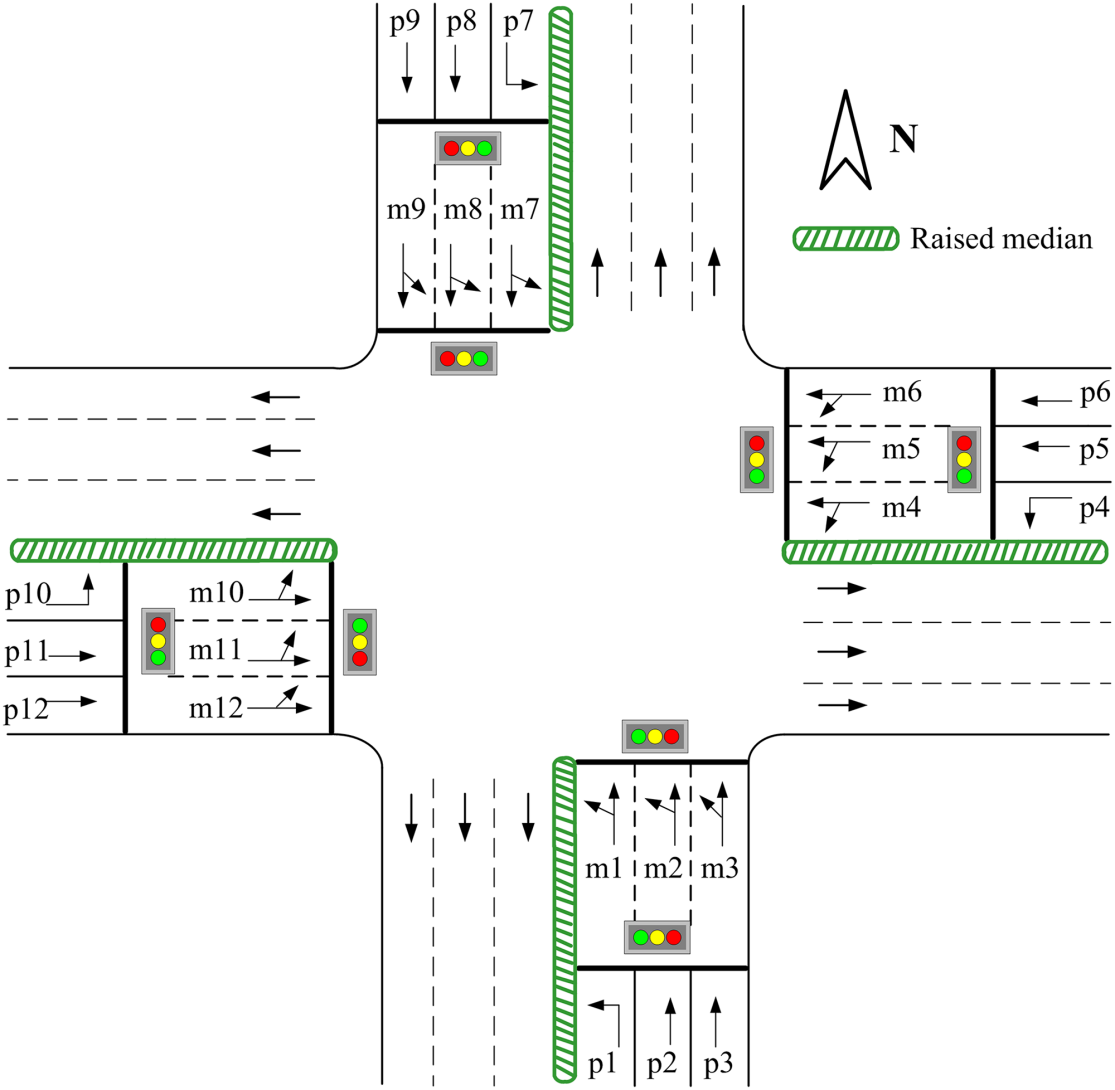
The intersection with pre-signals on all approaches.

Fig 4 shows a signalized intersection where the pre-signal is installed at each of the four approaches. The main signal is set at the first stop line beside the intersection for controlling left-turn and through vehicles within the sorting area, while the pre-signal installed at the upstream stop line aims to control the vehicles approaching the sorting area.

For example, there are three lanes at the south approach, named m1, m2, and m3. The number of lanes for the left-turn vehicles should not exceed that of the west exit, which is three as shown in Fig 4. Hence the left-turn vehicles can use either of the three lanes. However, if there are only two lanes at the west exit, the left-turn vehicles can only use m1 and m2. Since this study focuses on the traffic congestion mainly caused by the left-turn or through vehicles at the intersection, the right-turn vehicles are thus excluded from the subsequent analysis and modeling.

Under the phase swap sorting strategy, vehicles may be delayed and stay in the sorting area which reduces the intersection capacity and results in chaotic traffic movements. To address this concern, the end time of pre-signal’s green phase often precedes that of the main signal under the fixed-time control strategy, i.e. an offset is present. Since real-time information of vehicular movements is not accessible and speed differences exist between different vehicles, the offset is often calculated in a conservative manner, i.e. set as the maximum value of the travel time within the sorting area. Though this method can guarantee smooth traffic flow at the tandem intersection, the green phase of the main signal may not be fully used. Let the offset be 15 s, for example, and a vehicle needs 10 s to travel from the pre-signal stop line to the main signal, then 5 s of green time is wasted for the main signal. Considering that there are four main signals installed at the intersection, this amount of green time loss can significantly reduce the intersection capacity.

Adaptive control, on the other hand, can dynamically adjust the signal phasing according to the real-time vehicular movements monitored by the loop detectors installed at the approach. This type of control method can be applied to the tandem intersection to dynamically determine the green phase of the main signal given that the real-time vehicular information is provided by the loop detectors. As a result, both the vehicle delay in the sorting area and the waste of green time can be avoided, suggesting that the layout of loop detectors is fundamental to dynamic control.

**Fig 5:**
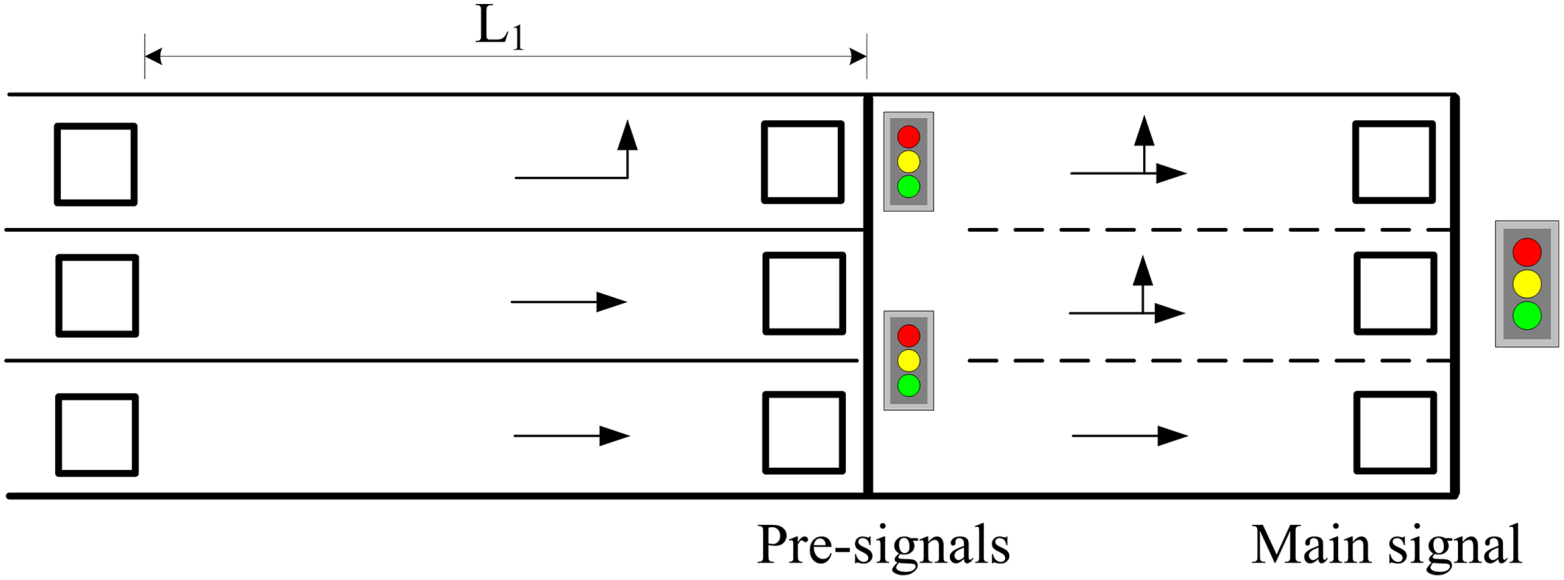
The layout scheme of loop detectors.

We set three groups of loop detectors at each approach of the intersection as shown in Fig 5. The first group is set L_1_ m upstream of the pre-signal stop line to monitor arrival vehicles. The method for determining L_1_ will be discussed in Subsection 2.3.2. The second group is set behind the pre-signal stop line that counts the number of vehicles in each lane entering the sorting area in each cycle. The third group is set behind the main signal stop line so that vehicles exiting the intersection during the green phase of each cycle are counted for each lane.

### Signal control algorithm

The phasing sequence illustrated in Fig 2 shows that the key parameters for signal control at the tandem intersection include the green time of the pre-signal, the green time of the main signal, and the offset between the pre-signal and the main signal. The cycle length does not matter since this study focuses on the adaptive control strategies, i.e. using real-time vehicular information provided by the loop detectors to check whether green extension is needed. Detailed discussions about each of the three parameters are presented below.

#### (1) Green time of main signal phase

Only when the number of vehicles counted at the pre-signal equals that at the main signal shall the green phase of the main signal end. Therefore, the end time of main signal’s green phase depends on the clear time of vehicles in the sorting area.

The start of main signal’s green time is determined by the end of the green time of the previous main signal. For example, in Fig 2, only when the green and amber lights of Mewl ends shall Msnl turns green.

Hence the green time of the main signal can hardly be optimized since it is related with the time that all the vehicles in the sorting area exit the intersection. Speed variations between different vehicles may lead to inaccurate prediction, but the green time of the main signal can still be predicted based on the queuing conditions in the sorting area as discussed in Section 2.3.

#### (2) Offset between main signal and pre-signal

The offset between the main signal and the pre-signal is of great importance to both the vehicle delay and stop frequency in the sorting area. For example, the offset between Msnl and Psnl as shown in Fig 2 may influence the left-turn vehicles from the south and north approaches. Fig 2 also shows that Psnl and Mewl turn green simultaneously. When the amber phase of Mewl ends, Msnl turns green. As a result, the offset between Msnl and Psnl equals the summation of the green and amber times of Mewl.

The above analysis results to the following conclusion: the offset between the main signal and the pre-signal for a specific approach equals the summation of the green and amber times of the previous main signal.

#### (3) Green times of pre-signal phases

We can see from Fig 2 that Psnl and Mewl turn green simultaneously, and that the time when Mewl turns green is determined by the end of Msnl’s amber light.

The end of Psnl’s green light is largely influenced by the traffic conditions at the approaching lanes. Specifically, green time may be wasted if no vehicle is detected in the approaching lanes but the signal still shows green. More green time allows more vehicles to enter the sorting area which essentially leads to a larger capacity and smaller average vehicle delay of the current phase, yet there may be some negative effects for the other phases. Therefore, the end of pre-signal’s green light needs to be optimized.

The detailed optimization method for the end of pre-signal’s green time is presented in the following Subsection.

### Development of the optimization model

#### Queuing process in the sorting area

**Fig 6:**
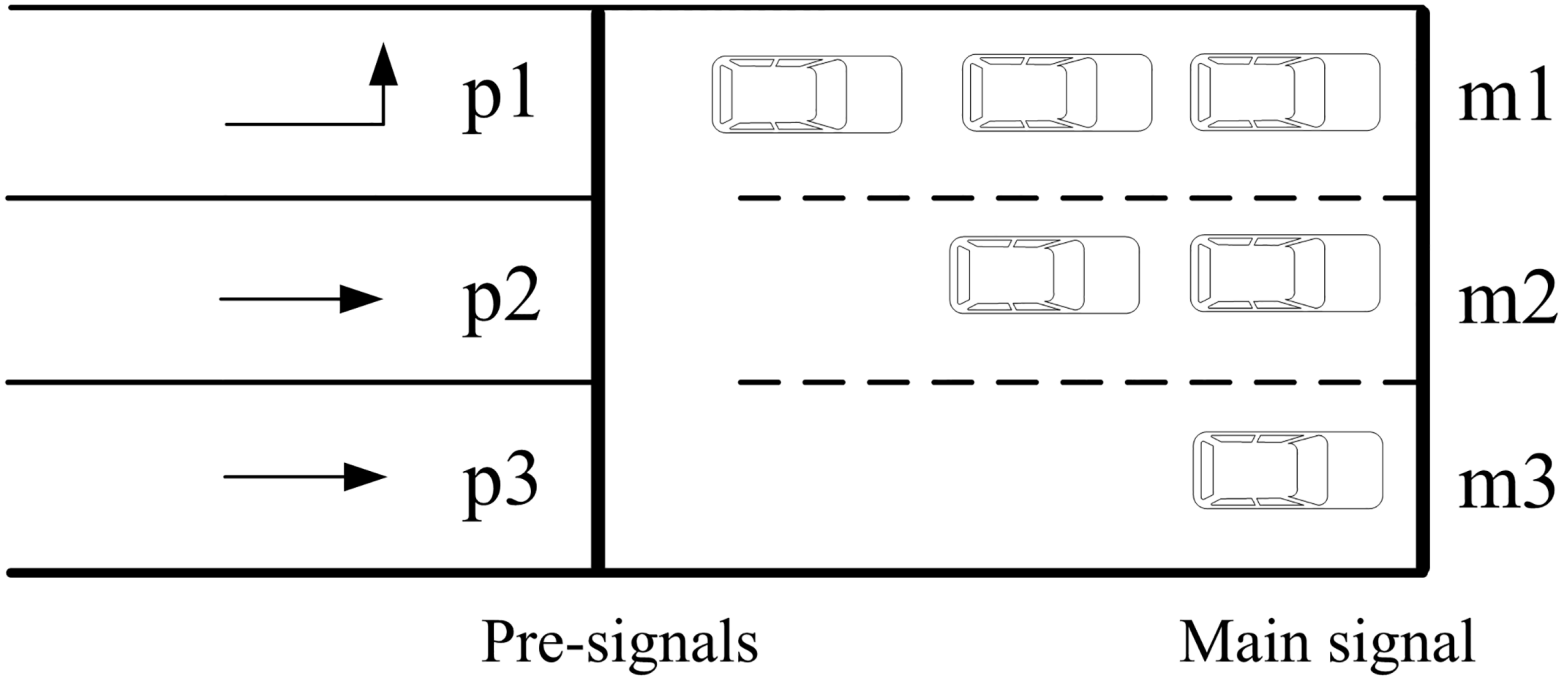
The queue status in sorting area.

The queuing process within the sorting area can affect the vehicle delay, the green time of the main signal, and the length of the sorting area. Previous studies assumed that vehicles entering the sorting area are evenly distributed across all the lanes. When 6 vehicles enter the sorting area, for example, each lane shall accommodate 2 vehicles. Nonetheless, when all the lanes in the sorting area are not fully occupied by queuing vehicles, the lane selection behavior may be affected the number of lane changing operations needed, the total travel time taken to exit the sorting area, etc. and gives rise to the stepped shape of the queuing process shown in Fig 6.

The lane m1 is the first priority for the left-turn vehicles because multiple lane changing operations are required to enter either m2 or m3, to which the drivers are often reluctant. Furthermore, the longer turning distance of m2 or m3 leads to a larger traveling time through the intersection. When the number of vehicles queuing in m1 exceeds that in m2 by Δ*N*_*L*_, the left-turn vehicles would enter m2. Though the lane changing is needed to enter m2, once the signal shows green the vehicles can exit the intersection faster. Similarly, when the number of vehicles queuing in m1 exceeds the number in m3 by 2Δ*N*_*L*_ and the number in m2 exceeds that in m3 by Δ*N*_*L*_, the left-turn vehicles may enter m3.

The above analysis assumes that m1, m2 and m3 are not fully occupied. If m1 is fully occupied, the following vehicles can only choose between m2 and m3. If both m1 and m2 are saturated, the following vehicles can only enter m3.

When the pre-signal turns to green, through vehicles can choose between m1, m2, and m3. Since vehicles keep entering the sorting area through p2, the lane changing behavior for vehicles moving in p3 can be quite difficult. Hence through vehicles in p2 may enter either m1 or m2, but those in p3 can only enter m3. Note that m2 remains the first priority for through vehicles in p2 till the number of vehicles in m2 exceeds that in m1 by Δ*N*_*L*_.

Determining Δ*N*_*L*_ is critical in establishing the vehicle queuing model in the sorting area. The pre-signal method has been applied in a number of cities in China such as Chengdu, Shanghai, Shenzhen, etc. Hence Δ*N*_*L*_ can be determined through field surveys. In contrast, we propose a method for determining Δ*N*_*L*_ using the data collected by the loop detectors. Taking the northbound approach in Fig 4 as an example, when the left-turn phase of the main signal turns green, vehicles queuing in the three lanes start to move through the intersection and hence, the loop detectors installed in the sorting area can count the exiting vehicles for each lane, denoted as *N*_*m*1_, *N*_*m*2_, and *N*_*m*3_. Assuming that each lane can accommodate up to *K*_*m*_ vehicles and are under-saturated, i.e. (*N*_*m*1_ < *K*_*m*_)&(*N*_*m*2_ < *K*_*m*_)&(*N*_*m*3_ < *K*_*m*_), then the following equations hold:

$$\Delta N_{L}(m_{1})=N_{m1}-N_{m2}$$ (1)

$$\Delta N_{L}(m_{2})=N_{m2}-N_{m3}$$ (2)

By collecting multiple pairs of Δ*N*_*L*_(*m*_1_) and Δ*N*_*L*_(*m*_2_), and calculating the average values denoted by $\Delta {\bar{N}}_{L}(m_{1})$ and $\Delta {\bar{N}}_{L}(m_{2})$, the final Δ*N*_*L*_ is expressed as

$$\Delta N_{L}=\text{Round}(\frac{\Delta {\bar{N}}_{L}(m_{1})+\Delta {\bar{N}}_{L}(m_{2})}{2})$$ (3)

where Round(·) is a rounding function.

In Eq 3, large vehicles such as trucks and buses are not considered. Special detectors can be employed to obtain the lengths of arrival vehicles when the large vehicle volume is high enough.

#### Planning horizon

**Fig 7:**
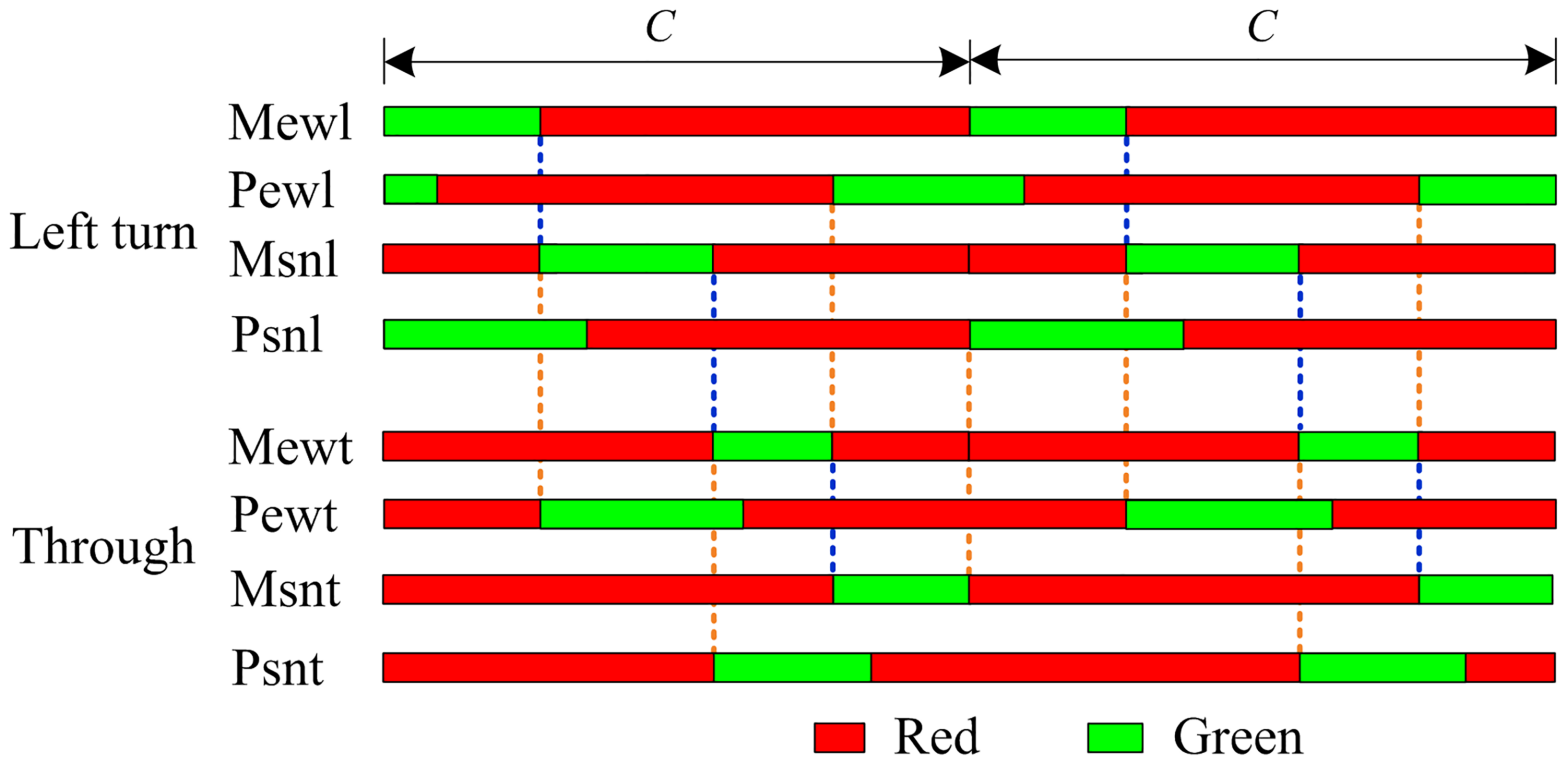
The intersection phasing scheme without intergreen time.

We take Psnl as an example to show the optimization of the end of green time. For modeling simplicity, the amber phase is excluded and the corresponding phase scheme is shown in Fig 7.

Let $t_{gewl}^{m}$ and ${\hat{t}}_{gewl}^{m}$ be the start and end of Mewl’s green time, and ${\hat{t}}_{gewl}^{p}$ be the end of Pewl’s green time. The start of Psnl’s green time, $t_{gsnl}^{p}$, equals $t_{gewl}^{m}$, and the start of Msnl’s green time, $t_{gsnl}^{m}$, equals ${\hat{t}}_{gewl}^{m}$. Assuming that the minimum and maximum green time of Psnl are $g_{psnl}^{\text{min}}$ and $g_{psnl}^{\text{max}}$ respectively, ${\hat{t}}_{gsnl}^{p}$ should satisfy the following inequality: $g_{psnl}^{\text{min}}\leq ({\hat{t}}_{snl}^{p}-t_{snl}^{p})\leq g_{psnl}^{\text{max}}$.

**Fig 8:**
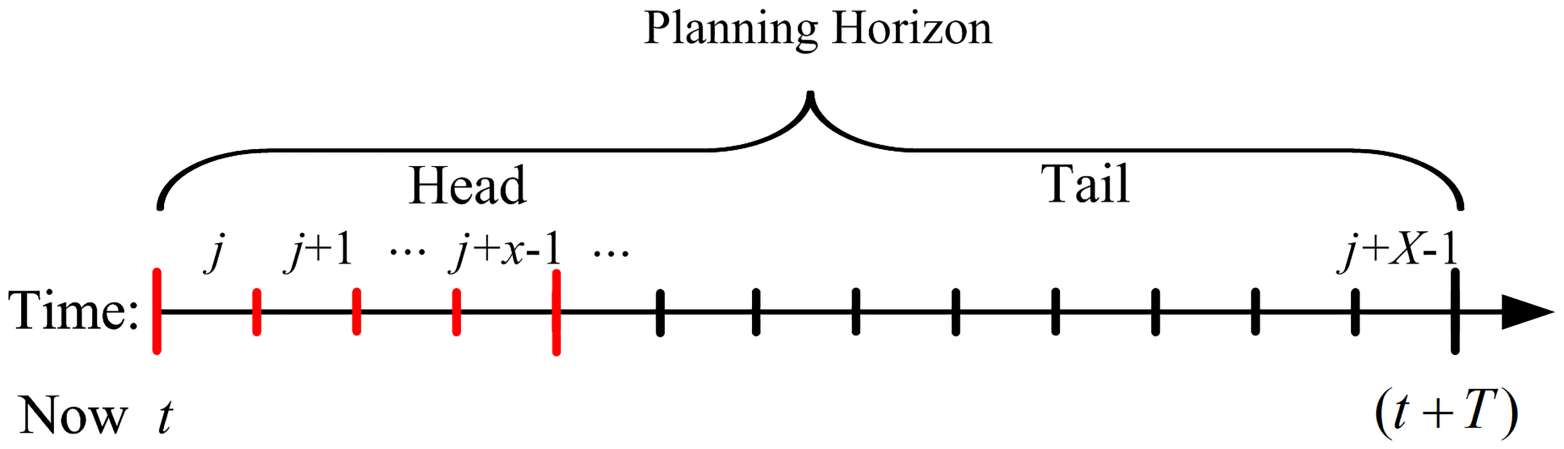
Division of planning horizon.

Inspired by the adaptive signal control method introduced in the OPAC system [18], we partition the time horizon into multiple intervals as shown in Fig 8. Each time interval (out of the *X* time intervals) has T s with Δ*t* s as the basic time unit. By the end of each time interval, the signal controller determines, taking into account the overall operation efficiency of traffic at the intersection during the next T s, the green phases of the pre-signal and the main signal for the next time interval. For example, the signal controller considers, at the beginning of the time interval *j*, the traffic flow in the next T s to determine the green phases of the main signal and the pre-signal in the time interval *j*. At the beginning of the time interval (*j* + 1), the signal controller re-optimizes, i.e. every optimization only applies to the current time interval.

Let the beginning of the time interval *j* be *t*. The planning horizon can be divided into two parts: Head section consisting of *x* time intervals and Tail section which has (*X*-*x*) time intervals. The following equation should hold:

$$x=\frac{L_{1}/V}{\Delta t}$$ (4)

where *L*_1_ (m) and *V* (m/s) are the distance and the average vehicle speed from the first group of loop detectors to the pre-signal stop line.

Eq 4 shows that *x* is the number of time intervals needed for vehicles to travel from the first group of loop detectors to the pre-signal stop line. Within the Head section, the signal controller can accurately predict the number of arrivals using the data provided by the first group of loop detectors. In the Tail section, however, the signal controller cannot obtain the accurate number of arrivals at the pre-signal and hence, can only predict using the historical data.

Due to the lane changing behavior of vehicles traveling between the first group of loop detectors and the pre-signal stop line, the number of arrivals counted by the detectors may not equal that at the stop line, particularly when *L*_1_ is quite large. Since a larger *L*_1_ leads to a larger discrepancy, we often set *L*_1_ to be any value between 80 to 100 m, as an integral multiple of (*V*·Δ*t*).

The span of the planning horizon, T, is critical to the signal control scheme. Normally a larger T allows more vehicles to be considered when the signal controller optimizes the signal timing. However, since the length of the Head section is fixed, a larger T also leads to a longer Tail section. Note that the traffic flow in the Tail section can only be predicted in an inaccurate manner that may result in certain degrees of predictive errors, which negatively affects the decision making of the signal controller. Hence, T should not be set extremely large. To this end, we set T as 40 s [19].

#### System state description

We use the queuing and states of signals to formulate the traffic state at the intersection. The queue length of p1, for example, is denoted by *W*_*p*_(1). Since there are totally *N*_*p*_ approaching lanes for the pre-signal, the queue lengths are expressed as the following vector:

$$W_{p}=\left[W_{p}(1),W_{p}(2),\cdots ,W_{p}(N_{p})\right]$$ (5)

Let *S*_*p*_(1) be the signal light state of p1. The signal states of the *N*_*p*_ approaching lanes for the pre-signal are thus expressed as the following vector:

$$S_{p}=\left[S_{p}(1),S_{p}(2),\cdots S_{p}(N_{p})\right]$$ (6)

$$\begin{array}{ll} S_{p}(n_{p}) & =\{\begin{array}{ll} 1 & \text{if signal is green for lane }n_{p}\\ \text{0} & \text{if signal is red for lane }n_{p} \end{array} \end{array}$$ (7)

To reduce the complexity of the subsequent optimization model, the amber light is excluded in both Fig 7 and Eq 7, i.e. only green and red lights are considered.

Similarly, let *N*_*m*_ be the number of lanes in the sorting area, *W*_*m*_(1) and *S*_*m*_(1) be the queuing and signal state of m1. The queuing lengths and signal state of the *N*_*m*_ approaching lanes in the sorting area are thus expressed as

$$W_{m}=\left[W_{m}(1),W_{m}(2),\cdots W_{m}(N_{m})\right]$$ (8)

$$S_{m}=\left[S_{m}(1),S_{m}(2),\cdots S_{m}(N_{m})\right]$$ (9)

$$\begin{array}{cc} S_{m}(n_{m}) & =\{\begin{array}{ll} 1 & \text{if signal is green for lane }n_{m}\\ \text{0} & \text{if signal is red for lane }n_{m} \end{array} \end{array}$$ (10)

Let *Q*_*p*_ be the number of arrivals in each lane at the pre-signal stop line, and *Q*_*m*_ be the number of vehicles in each lane within the sorting area:

$$Q_{p}=\left[Q_{p}(1),Q_{p}(2),\cdots Q_{p}(N_{p})\right]$$ (11)

$$Q_{m}=\left[Q_{m}(1),Q_{m}(2),\cdots Q_{m}(N_{m})\right]$$ (12)

The number of arrivals in each lane at the pre-signal can be monitored by the first group of loop detectors, or be predicted, whereas the number of vehicles entering the sorting area can be provided by the second group of loop detectors installed at the pre-signal. As per the queuing rules in the sorting area, the number of vehicles in each lane can be obtained.

Let *E*_*p*_ be the number of departures in each lane at the pre-signal stop line (note that in saturated conditions, *E*_*p*_ is smaller than *Q*_*p*_), and *E*_*m*_ be the number of vehicles in each lane leaving the sorting area:

$$E_{p}=\left[E_{p}(1),E_{p}(2),\cdots E_{p}(N_{p})\right]$$ (13)

$$E_{m}=\left[E_{m}(1),E_{m}(2),\cdots E_{m}(N_{m})\right]$$ (14)

*E*_*p*_ and *E*_*m*_ can be monitored by the loop detectors installed at the stop lines.

At the beginning of the time interval *j*+1, the number of queuing vehicles in *n*_*p*_ (at the pre-signal) and *n*_*m*_ (in the sorting area) are mathematically expressed as

$$\begin{array}{cc} l_{p}^{j+1}(n_{p})=l_{p}^{j}(n_{p})+Q_{p}^{j}(n_{p})-E_{p}^{j}(n_{p}) & 1\leq n_{p}\leq N_{p} \end{array}$$ (15)

$$\begin{array}{cc} l_{m}^{j+1}(n_{m})=l_{m}^{j}(n_{m})+Q_{m}^{j}(n_{m})-E_{m}^{j}(n_{m}) & 1\leq n_{m}\leq N_{m} \end{array}$$ (16)

Eqs 15 and 16 are the transition functions of the queuing states at the intersection.

#### Signal control decisions

Fig 7 shows that there are four phases for the main signal, and that only one can be green at a specific time point. Though the pre-signal also comprises of four phases, two of them are allowed to be green simultaneously, for example Pewl and Psnl. Since the start of Pewl’s green time precedes that of Psnl, we set Pewl as the first pre-signal phase whereas Psnl becomes the second.

Therefore, for the tandem intersection, the signal controller needs to make decisions at the beginning of each time interval for both the main signal and the pre-signal. The decision vectors for lanes controlled by the pre-signal and the main signal are expressed as:

$$u_{p}=\left[u_{p}(1),u_{p}(2),\cdots u_{p}(N_{m})\right]$$ (17)

$$u_{m}=\left[u_{m}(1),u_{m}(2),\cdots u_{m}(N_{m})\right]$$ (18)

At the beginning of the time interval *j*, the decision variable in the signal controller for the lane *n*_*p*_ is

$$\begin{array}{cc} u_{p}^{j}(n_{p}) & =\{\begin{array}{ll} 1 & \text{for signal switch}\\ \text{0} & \text{unchanged} \end{array} \end{array}$$ (19)

At the beginning of the time interval *j*, the decision variable in the signal controller for the lane *n*_*m*_ is

$$\begin{array}{cc} u_{m}^{j}(n_{m}) & =\{\begin{array}{ll} 1 & \text{for signal switch}\\ \text{0} & \text{unchanged} \end{array} \end{array}$$ (20)

The transition of signal state for the lane *n*_*p*_ is mathematically expressed as

$$S_{p}^{j}(n_{p})=(S_{p}^{j-1}(n_{p})+u_{p}^{j}(n_{p})){\text{mod}}_{2}$$ (21)

where mod_2_ is the modulo operator on 2.

The transition of signal light state for the lane *n*_*m*_ can also be expressed similar to Eq 21, and thus is not presented here.

*u*_*p*_ and *u*_*m*_ are decision variables that directly affect the operation efficiency of traffic at the intersection. To be specific, the decision variables mainly control the start and end of the green time for both the pre-signal and the main signal. By analyzing the phasing scheme shown in Fig 7 and the traffic operation at the tandem intersection, three rules are obtained:

**Rule 1:** The start of the main signal’s green time depends on the end of the green time from the previous phase.

For example in Fig 7, Mewl is the predecessor of Msnl and hence, only when the green time of Mewl ends shall Msnl turns green.

**Rule 2:** Only when the numbers of vehicles entering and exiting the sorting area become equal shall the green phase of the main signal end.

The number of vehicles entering or exiting the sorting area can be provided by the installed loop detectors. The green phase of the main signal should end once the two numbers become equal.

Rule 1 and 2 imply that it’s not needed to optimize the start and end of the main signal’s green time, and that no restriction is imposed on the maximum or minimum green time of the main signal. As a result, the signal controller only needs to make decisions based on the rules presented here.

**Rule 3:** The start of green of the phase *i* from the main signal is also the start of green of the pre-signal’s phase, which corresponds to phase *i*+1 from the main signal.

Fig 7 shows that when Msnl turns green, the pre-signal phase that corresponds to Mewt, i.e. Pewt, also turns green.

The above three rules indicate that we do not need to optimize the start and end of the main signal’s green time as well as the start of the pre-signal’s green time. Only the operation rules presented here are needed. For the end of the pre-signal’s green time, however, no rule is available.

More green time allowed by the pre-signal results in more vehicles entering the sorting area. Hence the main signal needs a longer green phase which, however, increases the vehicle delay in the other phases. On the other hand, less green time from the pre-signal leads to a reduced capacity of the green phase and the resulting increased vehicle delay.

The decision-making process of the signal controller at the beginning of the time interval *j* is presented below.

**Step 1:** Calculate the vector $l_{m}^{j}$.

**Step 2:** For the main signal phase *i* that shows green during the time interval *j*-1, if the number of vehicles queuing in each lane in the sorting area equals zero, all the vehicles have been cleared and thus, go to Step 4. Otherwise go to Step 3.

**Step 3:** Set *u*_*m*_ = 0, i.e. the main signal remains unchanged for all the lanes in the sorting area.

**Step 4:** At the beginning of the time interval *j*, the decision variables are set as 1 for the lanes in the sorting area controlled by the main signal phases *i* and *i*+1, i.e. the signal is changed. For the lanes controlled by the other phases from the main signal, the decision variables are set as 0, i.e. the signal remains unchanged. For the lanes controlled by the pre-signal phase that corresponds to the main signal phase *i*+2, the decision variables are set as 1, i.e. the signal turns green, and this phase becomes the second pre-signal phase.

**Step 5:** For the first pre-signal phase that shows green during time interval *j*-1, if the minimum green time is reached, go to Step 6. Otherwise go to Step 7.

**Step 6:** For the first pre-signal phase that shows green during the time interval *j*-1, if the maximum green time is reached, go to Step 8. Otherwise go to Step 9.

**Step 7:** For the lanes controlled by all the pre-signal phases except those involved in Step 4, the decision variables are set zero, i.e. the signal remains unchanged.

**Step 8:** The decision variables for the lanes controlled by the first pre-signal phase are set as 1, i.e. the signal is changed. The decision variables for the lanes controlled by the other pre-signal phases except those involved in Step 4 are set as 0, i.e. the signal remains unchanged.

**Step 9:** Use the optimization algorithm to optimize the decision variables for each pre-signal phase (except the pre-signal phases involved in Step 4).

Since the end of the main signal’s green time is later than that of the pre-signal, Step 4 and 8 cannot be performed simultaneously, i.e. the green phases do not end at the same time for the main signal and the pre-signal.

The above procedure shows that optimizing the decision variables for the pre-signal phase in Step 9 is most critical at the beginning of each time interval. With the objective of minimizing the average vehicle delay at the intersection, the detailed algorithm for optimizing the decision variables of the pre-signal phase is presented in Subsection 2.3.5.

#### Optimization of decision variables

At the beginning of the time interval *j*, the signal controller needs to make decisions taking into account the *X* time intervals within the planning horizon. The decision outcomes, however, are only applied to the time interval *j*. At the beginning of the time interval *j*+1, the signal controller repeats the decision-making process. Such a multi-stage decision-making process can be solved using dynamic programming.

The vehicle delay at the intersection consists of two parts: the delay experienced prior to entering the sorting area and the delay experienced within the sorting area. The first type of delay is introduced below.

During the time interval *j*, the total vehicle delay of the lane *n*_*p*_ is expressed as

$$D_{p}^{j}(n_{p})=[l_{p}^{j-1}(n_{p})+Q_{p}^{j-1}(n_{p})-E_{p}^{j-1}(n_{p})]\cdot \Delta \text{t}$$ (22)

Since there are *N*_*p*_ lanes controlled by the pre-signal, the total vehicle delay during the time interval *j* equals

$$D_{p}^{j}=\sum_{n_{p}=1}^{N_{p}}D_{p}^{j}(n_{p})$$ (23)

where $D_{p}^{j}$ is often referred to as the one step cost function in dynamic programming. Since there are *X* time intervals in the planning horizon, the total vehicle delay of all the lanes controlled by the pre-signal equals:

$$\begin{array}{cc} J_{p}=\sum_{k=j}^{j+X-1}\gamma^{k-j}D_{p}^{k} & 0<\gamma \leq 1 \end{array}$$ (24)

In Eq 24, γ is a discount factor and it ranges from 0 to 1.0. It reflects the system preference to the one step costs in different steps. If γ equals 1.0, then *J*_*p*_ is the sum of one step costs of the steps from *j* to (*j*+*X*-1) and the system gives equal weight to each step. Otherwise *J*_*p*_ is the sum of discount costs of the steps.

Assuming $D_{p}^{1}(n_{p})=0$, the number of queuing vehicles at the beginning of each time interval can be obtained using Eq 15. How to determine $Q_{p}^{k}(n_{p})$ and $E_{p}^{k}(n_{p})$ is critical in Eqs 22–24, where *k* denotes the number of the time interval. $E_{p}^{k}(n_{p})$ is the number of vehicles exiting the lane *n*_*p*_ during the time interval *k*. At the beginning of the time interval *k*+1, the signal controller can obtain $E_{p}^{k}(n_{p})$ directly from the loop detectors installed at the pre-signal stop line. The method for obtaining $Q_{p}^{k}(n_{p})$ is discussed below.

**(i) when** *j* ≤ *k* ≤ (*j* + *x* − 1)

When *j* ≤ *k* ≤ (*j* + *x* − 1), $Q_{p}^{k}(n_{p})$ can be obtained using the number of arrivals provided by the first group of loop detectors. The time when the vehicle arrives at the pre-signal stop line equals the time when the vehicle reaches the first group of loop detectors plus the travel time between the detectors and the stop line.

**(ii)when** (*j* + *x*) ≤ *k* ≤ (*j* + *X* − 1)

When (*j* + *x*) ≤ *k* ≤ (*j* + *X* − 1), the number of time intervals *k* and *j* is larger than *x*. Since *x* time intervals are required for vehicles moving from the first group of loop detectors to the pre-signal stop line, the signal controller cannot obtain the real number of arrivals from the detectors. Rather, predictions are needed.

By using the algorithm introduced in the OPAC system [18], the number of arrivals in the lane *n*_*p*_ during the time interval *k* is computed based on historical data. The traffic flow rate (pcu/s) in lane *n*_*p*_ can be obtained using the vehicle count during the last five minutes provided by the first group of loop detectors. Hence $Q_{p}^{k}(n_{p})$ equals the traffic flow rate multiplied by Δ*t*.

The vehicle delay experienced within the sorting area is introduced next.

During time interval *j*, the total vehicle delay of lane *n*_*m*_ in the sorting area is expressed as:

$$D_{m}^{j}(n_{m})=[l_{m}^{j-1}(n_{m})+Q_{m}^{j-1}(n_{m})-E_{m}^{j-1}(n_{m})]\cdot \Delta \text{t}$$ (25)

Since there are *N*_*m*_ lanes in the sorting area, the total vehicle delay during the time interval *j* equals:

$$D_{m}^{j}=\sum_{n_{m}=1}^{N_{m}}D_{m}^{j}(n_{m})$$ (26)

Hence the total vehicle delay in the sorting area within the planning horizon equals

$$\begin{array}{cc} J_{m}=\sum_{k=j}^{j+X-1}\gamma^{k-j}D_{m}^{k} & 0<\gamma \leq 1 \end{array}$$ (27)

When the green phase of the main signal ends, the number of queuing vehicles in each of the controlled lanes equals zero. The number of queuing vehicles at the beginning of each time interval can be obtained using Eq 16. How to determine $Q_{m}^{k}(n_{m})$ and $E_{m}^{k}(n_{m})$ is critical in Eqs 25–27. $E_{m}^{k}(n_{m})$ is the number of vehicles exiting the lane *n*_*m*_ during the time interval *k*. At the beginning of the time interval *k*+1, the signal controller can obtain $E_{m}^{k}(n_{m})$ directly from the loop detectors installed at the main signal stop line. The method for obtaining $Q_{m}^{k}(n_{m})$ is discussed below.

$Q_{m}^{k}(n_{m})$ is the number of vehicles entering the sorting area through the lane *n*_*m*_ during the time interval *k*. It can be obtained using the vehicle count data provided by the loop detectors. Then the number of vehicles entering the sorting area in each lane can be derived using the queuing strategy introduced in Section 2.3.1.

#### (i) All the vehicles entering the sorting area during the time interval *k* are left-turn vehicles

Let *n*_*m*1_, *n*_*m*2_, and *n*_*m*3_ be the three lanes in the sorting area as shown in Fig 4. Note that *n*_*m*3_ is the rightmost lane, and that *n*_*m*_ used in Eq 25 can be either of the three. During the time interval *k*, when the left-turn phase of the pre-signal shows green and the through phase shows red, only left-turn vehicles enter the sorting area. Let *n*_*p*_ be the left-turn lane behind the pre-signal stop line. Left-turn vehicles entering through the lane *n*_*p*_ will queue in the sorting area according to the queuing rule introduced in Section 2.3.1.

The length of a single time interval, Δ*t*, is often set between 3 and 5 seconds. Even if the vehicles queuing behind the pre-signal stop line are discharged at the saturation flow rate, only a maximum of three vehicles can enter the sorting area through the lane *n*_*p*_ during the time interval *k*. Let $Q_{p}^{k}(n_{p})$ be the number of entering vehicles and then, the number of detected vehicles at the stop line in the lane *n*_*p*_ can only have four options for the time interval *k*, i.e. the integers from zero to three.

**Case I:** $Q_{p}^{k}(n_{p})=0$

No vehicle enters the sorting area during time interval *k*, $Q_{m}^{k}(n_{m1})=Q_{m}^{k}(n_{m2})=Q_{m}^{k}(n_{m3})=0$.

**Case II:** $Q_{p}^{k}(n_{p})=1$

At the beginning of the time interval *k*, the initial queuing lengths of the lanes *n*_*m*1_, *n*_*m*2_, and *n*_*m*3_ are denoted by $l_{m}^{k}(n_{m1})$, $l_{m}^{k}(n_{m2})$, and $l_{m}^{k}(n_{m3})$. No queue spillback occurs in the sorting area. Hence we build the following two parameters:

$$A_{1}=l_{m}^{k}(n_{m1})-l_{m}^{k}(n_{m2})$$ (28)

$$A_{2}=l_{m}^{k}(n_{m2})-l_{m}^{k}(n_{m3})$$ (29)

If *A*_1_ ≥ *A*_2_ ≥ Δ*N*_*L*_, the left-turn vehicle entering the sorting area chooses the lane *n*_*m*2_, $Q_{m}^{k}(n_{m2})=1$ and $Q_{m}^{k}(n_{m1})=Q_{m}^{k}(n_{m1})=0$

If *A*_2_ > *A*_1_ ≥ Δ*N*_*L*_, the left-turn vehicle enters the lane *n*_*m*3_ in the sorting area, $Q_{m}^{k}(n_{m3})=1$ and $Q_{m}^{k}(n_{m1})=Q_{m}^{k}(n_{m2})=0$.

For all the other scenarios, the left-turn vehicle enters the sorting area through the lane *n*_*m*1_, $Q_{m}^{k}(n_{m1})=1$ and $Q_{m}^{k}(n_{m2})=Q_{m}^{k}(n_{m3})=0$.

**Case III:** $Q_{p}^{k}(n_{p})=2$

The first left-turn vehicle chooses the lane according to Case II.

Let ${\vec{l}}_{m}^{k}(n_{m1})$, ${\vec{l}}_{m}^{k}(n_{m2})$, and ${\vec{l}}_{m}^{k}(n_{m3})$ be the initial queuing lengths of the lanes *n*_*m*1_, *n*_*m*2_, and *n*_*m*3_ when the second left-turn vehicle enters the sorting area. We re-build the parameters A_1_ and A_2_ using Eqs 28 and 29, based on which the lane selection behavior of the second left-turn vehicle is determined. The detailed discussion is not repeated here.

**Case IV:** $Q_{p}^{k}(n_{p})=3$

Similarly, the first two left-turn vehicles choose the lanes according to Case III. By re-building the parameters A_1_ and A_2_, the lane selection behavior of the third left-turn vehicle is determined. Note that the number of vehicles entering each lane is monitored.

#### (ii) All the vehicles entering the sorting area during the time interval *k* are through vehicles

During the time interval *k*, when the left-turn phase of the pre-signal shows red and the through phase shows green, only through vehicles enter the sorting area. Let *n*_*p*2_ and *n*_*p*3_ be the two through lanes behind the pre-signal stop line as shown in Fig 4. Note that *n*_*p*3_ is the rightmost lane.

Based on the queuing rule introduced in Section 2.3.1, through vehicles entering the sorting area from the lane *n*_*p*3_ do not change lane, i.e. $Q_{m}^{k}(n_{m3})=E_{p}^{k}(n_{p3})$.

The through vehicles in the lane *n*_*p*2_, the number of which equals $E_{p}^{k}(n_{p2})$, can choose between *n*_*m*1_ and *n*_*m*2_ in the sorting area. Let $l_{m}^{k}(n_{m1})$ and $l_{m}^{k}(n_{m2})$ be the initial queuing lengths of *n*_*m*1_ and *n*_*m*2_ at the beginning of the time interval *k*. Note that *n*_*m*2_ is closest to *n*_*p*2_. Hence the following parameter, A_3_, is built:

$$A_{3}=l_{m}^{k}(n_{m2})-l_{m}^{k}(n_{m1})$$ (30)

When *A*_3_ ≤ Δ*N*_*L*_, the first through vehicle moving in the lane *n*_*p*2_ enters *n*_*m*2_ in the sorting area; otherwise the lane *n*_*m*1_ is selected.

The lane selection behavior of through vehicles when entering the sorting area is also dependent on Case I-IV, and thus is not repeated further.

The vehicle delay at the intersection consists of two parts: the delay experienced prior to entering the sorting area and the delay experienced within the sorting area. Hence with the objective of minimizing the total vehicle delay at the intersection during the planning horizon, the end of the pre-signal’s green time is optimized by the following minimization program:

$$\begin{array}{cc} \text{min }J=J_{p}+J_{m}=\sum_{k=j}^{j+X-1}\gamma^{k-j}(D_{p}^{k}+D_{m}^{k})=\sum_{k=j}^{j+X-1}\gamma^{k-j}D^{k} & 0<\gamma \leq 1 \end{array}$$ (31)

*J* is the system value over the planning horizon. Eq 31 is a typical dynamic programming problem, which determines the decision variables of the pre-signal from the signal controller for the next *X* time intervals. Let $u_{p}^{*}$ be the optimized decision vector, then $u_{p}^{*}=\text{arg }(\text{min }J)$ which has *X* elements.

A number of studies have focused on the solution algorithms for Eq 31, e.g. the OPAC system that uses the classical backward induction algorithm. To improve the speed performance of the optimization model, Cai et al. [19] proposed a solution algorithm based on the approximate dynamic programming. In this paper, the classical backward induction algorithm is employed to solve the dynamic programming problem. The procedure is summarized as follows.

As for the planning horizon shown in Fig 8, the system value at time interval (*j*+*X*-1) equals:

$$J(j+X-1)=\text{min}\{D_{p}^{j+X-1}(l_{p}^{j+X-1},S_{p}^{j+X-1},u_{p}^{j+X-1})+D_{m}^{j+X-1}(l_{m}^{j+X-1},S_{m}^{j+X-1},u_{m}^{j+X-1})\}\cdot \gamma^{X-1}$$ (32)

In Eq 32, ***l*** and ***S*** are vectors of intersection traffic state variables. ***u*** is the vector of decision variables. The optimal decision variable vector $u*=(u_{p}^{*},u_{m}^{*})$ can minimize *J*(*j* + *X* − 1).

The system value from time interval (*j*+*X*-2) to (*j*+*X*-1) equals:

$$\begin{array}{cc} J(j+X-2) & =\begin{array}{l} \text{min}\{D_{p}^{j+X-2}(l_{p}^{j+X-2},S_{p}^{j+X-2},u_{p}^{j+X-2})+D_{m}^{j+X-2}(l_{m}^{j+X-2},S_{m}^{j+X-2},u_{m}^{j+X-2})\}\cdot \gamma^{X-2}\\ +J(j+X-1) \end{array} \end{array}$$ (33)

Similarly, the system value from time interval *k* to (*j*+*X*-1) equals:

$$J(k)=\text{min}\{D_{p}^{k}(l_{p}^{k},S_{p}^{k},u_{p}^{k})+D_{m}^{k}(l_{m}^{k},S_{m}^{k},u_{m}^{k})\}\cdot \gamma^{k-j}+J(k+1)$$ (34)

Then the system value from time interval *j* to (*j*+*X*-1) (the whole planning horizon) can be deduced:

$$J(j)=J=\text{min}\{D_{p}^{j}(l_{p}^{j},S_{p}^{j},u_{p}^{j})+D_{m}^{j}(l_{m}^{j},S_{m}^{j},u_{m}^{j})\}\cdot \gamma^{1}+J(j+1)$$ (35)

Based on the above algorithm, the optimal decision variables for pre-signals at each time interval over the planning horizon can be obtained. Note that *u*_m_ denote the decision variable vector for mail signals, thus it is also added in the solution algorithm. However, we don’t need to optimize it because the signal controller can automatically determine its optimal value according to the phasing scheme.

## Case studies

The proposed method is coded and tested in MATLAB. The testing scenario and the parameter setting are discussed in Section 3.1, while the comparative results are presented in Section 3.2.

### Test plans

The proposed adaptive control method, named Plan I, is compared with the fixed-time control method, named Plan II. We use the four-approach intersection with fully-installed pre-signals as the test object, shown in Fig 4.

In Plan I, each approach to the intersection has three lanes. Δ*N*_*L*_ is set as 2 pcu and the average vehicle speed is 10 m/s. The saturation flow rate of each lane is 1800 pcu/h and Δ*t* is 4 s [19]. Hence the planning horizon can be divided into ten time intervals, in which only maximum 2 pcu can exit (saturation flow rate equals 0.5 pcu/s). *L*_1_ is set 80 m. The head section in the planning horizon includes 2 time intervals while the tail section has 8. The discount factor, γ, is set as 0.6. The minimum and maximum green times of the left-turning main signal phases are 10 s and 40 s respectively. The minimum and maximum green times of the through main signal phases are 15 s and 40 s respectively. Within the maximum green time and saturation flow rate, 20 vehicles can depart from the sorting area from one approaching lane. Assuming the space headway under the queuing condition is 7 m, thus the distance between the two stop lines is set as 140 m.

To objectively evaluate the proposed dynamic control method for the tandem intersection, three traffic scenarios are used as shown in Table 1. Scenario 1 is the congested traffic state, Scenario 3 is the free-flow traffic state, and somewhere in between is the traffic state of Scenario 2. The arrivals of the left-turn and through vehicles (right-turn excluded) are assumed to follow a Poisson distribution. Only the green and red phases (amber excluded) are considered for decision making.

**Table 1:** The traffic volumes in three different scenarios (Unit: pcu/h).

Directions		Scenario 1	Scenario 2	Scenario 3
East leg	Left	600	400	200
	Through	1200	800	400
West leg	Left	600	400	200
	Through	1200	800	400
South leg	Left	600	400	200
	Through	1200	800	400
North leg	Left	600	400	200
	Through	1200	800	400

Under Plan II the intersection executes fixed-time control mode, thus the timing plans of all scenarios should be calculated, which are displayed in Table 2.

**Table 2:** Signal timing plans for Plan II under different scenarios (Unit: s).

Signal phases	Scenario 1	Scenario 2	Scenario 3
Mewl	22	16	13
Msnl	34	35	25
Mewt	40	34	26
Msnt	40	34	26
Pewl	47	35	24
Psnl	41	36	23
Pewt	59	54	36
Psnt	65	53	37

To compare the two control schemes, the average vehicle delay (AVD) and the maximum queue length (MQL) behind the pre-signal stop line are used as the main performance measures. The simulation is run for each scenario for 3600 s.

### Results and comparison

Table 3 summarizes the AVD (unit: s) and MQL (unit: m) under three different scenarios, which are further subdivided for the left-turn vehicles, through vehicles, and the intersection as a whole. Further analysis of the data shown in Table 3 is presented below. Please note that the MQL of the intersection equals the maximum value of left-turn MQL and through MQL.

**Table 3:** Simulation results of the two control plans under three different scenarios.

Directions	Indexes	Plan I			Plan II		
		Scenario 1	Scenario 2	Scenario 3	Scenario 1	Scenario 2	Scenario 3
Left turns	AVD	82.8	58.4	33.7	83.7	66.3	41.6
	MQL	138	78	24	143	84	28
Through	AVD	109.9	66.9	34.3	113.6	79.4	43.8
	MQL	262	139	56	270	148	63
Intersection	AVD	100.9	64.1	34.1	103.6	75.1	43.1
	MQL	262	139	56	270	148	63

#### (1) Plan I outperforms Plan II in all scenarios, the merits of Plan I increase with the decrease of intersection traffic load

From Table 3 we can find that the AVD and MQL under Plan I are all smaller than that under Plan II. In scenario 1 the intersection is in saturated state, thus the values of AVD and MQL under the two plans are close to each other. For example, in scenario 1 the intersection AVD and MQL under Plan I are lower than that under Plan II by 2.64% and 2.96% respectively.

**Fig 9:**
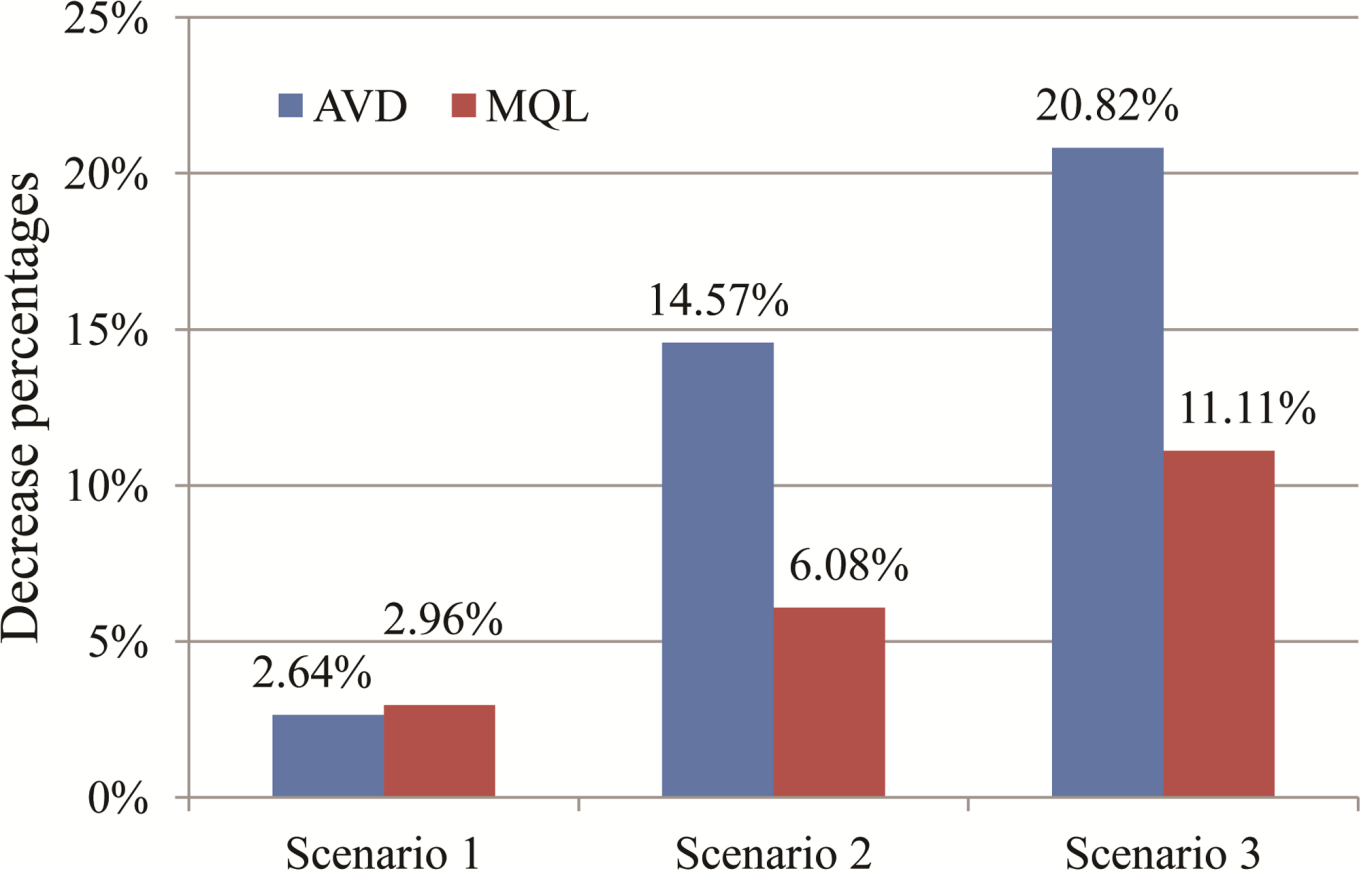
Decrease percentages in AVD and MQL of Plan I.

The advantages of Plan I over Plan II become more significant with the decrease of intersection traffic load. In scenario 2, the intersection AVD and MQL under Plan I are lower than that under Plan II by 14.57% and 6.08% respectively. In scenario 3, the decrease ratios of AVD and MQL reach 20.82% and 11.11%. Fig 9 displays the decrease ratios of AVD and MQL in three scenarios as compared with Plan II.

For through vehicles or left-turning vehicles, their AVD and MQL are also smaller than that under Plan II. Taking scenario 2 as an example, the AVD of left-turning vehicles under Plan I is 58.4s. Under Plan II, the AVD reaches 66.3s. In other scenarios the same trends can also be found, which indicates the effectiveness of Plan I.

#### (2) The developed adaptive control methods can accommodate the traffic flow fluctuations

The reason that Plan I outperforms Plan II is because the developed adaptive control methods in this study can accommodate the traffic flow fluctuations. Here the fluctuations refer to two aspects. The first aspect is that arrival vehicles follow a Poisson distribution, rather than a uniform distribution. The second aspect is that the vehicle speed in the sorting area is not a constant. When conducting simulations, the vehicle speed in the sorting area follows Normal distribution within the interval [9, 11]. In such condition, a larger offset should be set between the pre-signal and the main signal in Plan II, to avoid that vehicles are delayed in the sorting area. The timing plan may lead to the waste of green time. Moreover, more green time will be wasted with the increase of fluctuation range, which results in the increase of AVD and MQL under Plan II.

Under Plan I, the signal controller judges whether to end current green light of the main phase according to real-time traffic information. When the number of vehicles departed from pre-signal stop line equals that departed from main signal stop line, the green light of the main phase will be terminated immediately. In such way the waster of green time can be avoided.

## Conclusions

By increasing the number of available lanes for either the left-turn or through vehicles during the green phase, the method of pre-signal control can significantly improve the capacity of the intersection and reduce the traffic congestion. Therefore, this paper focuses on a four-approach tandem intersection with fully-installed pre-signals. Based on the phase swap sorting strategy, a queuing model for vehicles entering the sorting area is developed and a real-time signal control method is proposed using dynamic programming. The control method is demonstrated through numerical tests. Several conclusions have been reached as follows:

1. By installing loop detectors at the intersection to obtain real-time information of traffic flow and setting the operation rules for the main signal and the pre-signal, vehicles are no longer delayed in the sorting area and hence, the robustness of the signal control is increased.
2. Vehicles entering and queuing in the sorting area are not uniformly distributed across various lanes. There are certain degrees of preference which influence the green time of the main signal and the length of the sorting area.
3. For the pre-signal and the main signal, an appropriate configuration of the start and end of the green phase can improve the operation efficiency of traffic. However, only the end of pre-signal’s green time can be optimized. All the other time points can be obtained directly from the corresponding phase sequence.
4. The proposed method for dynamic signal control can significantly reduce the average vehicle delay and maximum queue length. The largest reduction can reach 20.82%.

## Supporting information

S1 File Available traffic data used in this study. (XLSX)
